# Membrane inlet mass spectrometry method for food intake impact assessment on specific volatile organic compounds in exhaled breath

**DOI:** 10.1007/s00216-022-04168-3

**Published:** 2022-06-21

**Authors:** Milena Jakšić, Andrea Mihajlović, Djordje Vujić, Stamatios Giannoukos, Boris Brkić

**Affiliations:** 1 BioSense Institute, University of Novi Sad, Dr Zorana Djindjića 1, 21000 Novi Sad, Serbia; 2 Department of Chemistry and Applied Biosciences, ETH Zurich, HCI D 317, Vladimir-Prelog-Weg 3, CH-8093 Zurich, Switzerland

**Keywords:** Breath, VOCs, MIMS, Portable, Food impact

## Abstract

This research work describes the development of a novel bioanalytical method for the assessment of food impact on selected exhaled breath volatile organic compounds (VOCs) using a fast and portable screening VOC prototype sensor based on membrane inlet mass spectrometry (MIMS). Method and sensor prototype functionality was verified by obtaining good response times, linearity in the examined concentration ranges, and sensitivity and repeatability for several breath VOCs—acetone, ethanol, n-pentane, and isoprene. A new VOC sensor prototype was also proven to be sensitive enough for selected breath VOC quantification with limits of detection at low part per billion (ppb) levels—5 ppb for n-pentane, 10 ppb for acetone and ethanol, and 25 ppb for isoprene. Food impact assessment was accomplished by tracking the levels of acetone, ethanol, n-pentane, and isoprene in exhaled breath samples collected from 50 healthy participants before the meal and 60 min and 120 min after the meal. For acetone, isoprene, and n-pentane, a larger impact was noticed 120 min after the meal, while for ethanol, it was after 60 min. Obtained VOC levels were in the expected concentration ranges. Mean values at all time points were ~ 500–900 ppb for acetone and ~ 400–600 ppb for ethanol. Most of the results for n-pentane were below 5 ppb, but the mean value for those which were detected was ~ 30 ppb. Along with samples, data about participants’ lifestyle were collected via a short questionnaire, which were compared against obtained VOC levels in order to reveal some significant correlations between habits of participants and their breath VOC levels.

**Graphical abstract:**

Portable MS: monitoring of food impact on the levels of selected VOCs from exhaled breath

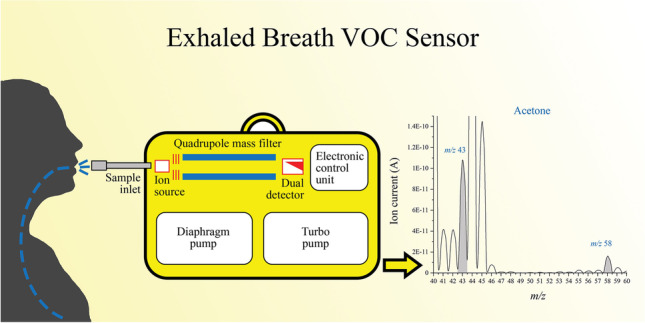

## Introduction

Breath research is an old research field, but since the 2000s, it has become truly fruitful [1–3]. According to the latest research findings referring volatile organic compounds (VOCs) from all bodily fluids, 1488 VOCs are found in human exhaled breath [4]. It is estimated that the presence of approximately 99% of the breath VOCs is individually dependent—on the living or working environment, personal lifestyle habits, diet, etc., which gives that only 1% of breath VOCs could serve as specific biomarkers for certain diseases or health conditions [5]. Moreover, breath as an analytical matrix is very changeable and unstable, per se. It is very hard to accomplish standardization of breath analysis, since there are many factors which contribute to the uncertainty of an analytical method for breath VOC determination, such as sampling methodology, sample storage, and choice of analytical equipment [3, 6–9]. As there are many tackle points, modern science can offer many solution possibilities as well [10]. In addition to diagnosing various diseases, breath analysis is also used to monitor the general condition of the body and determine the influence of various factors on it—therefore, diet and physical activity are inevitable parameters whose impact would be of interest to be monitored in this way [11]. This area in breath analysis has been less studied in relation to attempts to identify certain diseases, but there are certainly studies that have started research in this area [12–19].

Dietary habits and the impact of food on metabolism as well as the body as a whole are a growing concern of modern society. Food metabolism involves numerous processes that involve many structurally different metabolites. Some of these metabolites are volatile and tend to pass from the blood into the air in the lungs, reaching the exhaled breath [13]. That makes exhaled breath an instant reflection of the current state of the organism. Considering the fact that breath sampling is non-invasive, it is logical why more and more scientists are trying to seize and exploit all that potential which indisputably lies in the development of rapid, screening diagnostic tests for breath analysis [20, 21].

In the current study, analytes of interest are VOCs that are related to the metabolism of the main constituents of food—carbohydrates, proteins, fats: acetone, ethanol, isoprene, and n-pentane. Acetone is formed as a product of the oxidation of fatty acids in the state of starvation [20], and its elevated level has also been proven in people with diabetes [3, 4, 22]. Isoprene is a by-product of cholesterol synthesis and may indicate changes in fat metabolism [21, 22]. Along with acetone, it can be an indicator of diabetes, i.e., carbohydrates metabolism disorder [3, 23, 24]. There is also a link between elevated isoprene levels and a recent period of increased physical activity [3, 4, 25]. Tests for the presence of ethanol in exhaled breath have already been designed for traffic safety purposes, but it is known that ethanol, in addition to being ingested through alcoholic beverages, is also produced as a product of the activity of gastrointestinal bacteria and bacteria in the oral cavity [3, 26]. Another compound of interest is n-pentane, whose elevated level in exhaled breath is related to oxidative stress, because it is formed during lipid peroxidation of ω-3 and ω-6 fatty acids [27, 28]. Its increased level is related with mental and physical stress [3, 4, 24]. Characteristic chemical and physical properties of the selected analytes are listed in Table 1 [29–32].

**Table 1:** Overview of the general properties of selected VOCs [29–31]

Volatile organic compound	Acetone	Ethanol	n-Pentane	Isoprene
CAS no	67–64-1	64–17-5	109–66-0	78–79-5
Molecular formula	C_3_H_6_O	C_2_H_6_OH	C_5_H_12_	C_5_H_8_
Molecular weight (g/mol)	58.08	46.07	72.15	68.12
Most abundant electron impact (EI) mass fragments according to NIST database (*m*/*z*)	43, 58	29, 31, 45	43, 41, 42	67, 68
Vapor pressure (mmHg at 25 °C)	231	59.3	514	550
Boiling point (°C)	56.08	78.2	36.06	34.07
Henry’s Law constant (atm-cu m/mole)	3.97 × 10^−5^	5 × 10^−6^	1.25	7.7 × 10^−2^

Spectroscopic techniques are predominantly used for breath VOC analysis. Among numerous analytical techniques in this area, gas chromatography coupled to mass spectrometry (GC–MS) is considered as the gold standard in breath analysis [10, 12]. Specifically in the assessment of diet-related breath VOCs, GC–MS is commonly coupled with thermal desorption (TD) [12, 13, 34–36] and solid-phase microextraction (SPME) [18] as preconcentration techniques. In addition to these well-established analytical techniques, any progress towards facilitating and simplifying technical requirements is of great importance, in order to make new diagnostic methods as accessible as possible. Recent advances involve ambient ionization approaches coupled to various mass analyzers. Characteristic examples are proton transfer reaction MS (PTR-MS) [22], selected ion flow tube MS (SIFT-MS) [16], and secondary electrospray ionization high-resolution MS (SESI-HRMS) [37]. There are also attempts to avoid strict laboratory requirements and to enable more affordable, fast, and real-time breath VOC analysis. Such requirements employ in-field analytical techniques such as ion mobility spectrometry (IMS) [38], aspiration ion mobility spectrometry (AIMS) [17], field asymmetric ion mobility spectrometry (FAIMS) [21, 39], multicapillary column ion mobility spectrometry (MCC IMS) [19], and membrane-inlet MS (MIMS) [40]. Ultraviolet and infrared spectroscopy are also involved in breath analysis, but with less sensitivity and selectivity than mass spectroscopy techniques [21]. Beside spectroscopic techniques, there is great potential in the rapidly growing field of sensors, among which metal oxide semiconductors (MOS) are currently the most investigated and employed in this research area [3].

This research work aims to enhance breath research by employing a high-throughput membrane-inlet mass spectrometer with a single quadrupole mass analyzer with real-time monitoring capabilities. This is the first time that such instrument is utilized for the assessment of the food impact in the exhaled breath VOC research, to the authors’ best knowledge. Membrane-introduction mass spectrometry is a simple analytical technique. Its principle lies in the process of pervaporation, where analytes in the gas phase migrate through a semi-permeable membrane [41]. This migration through the membrane takes place in three steps: selective adsorption of the analyte on the membrane, diffusion of the analyte through the membrane, and desorption of the analyte from the membrane into the vacuum system of the mass spectrometer where it reaches the mass analyzer. MIMS is a technique that has been established in the 1970s. From that time, it has undergone many transformations, both in the development of membrane inlets and in the use of various mass analyzers [42]. As a consequence of the development of the instrumental part of the technique, the range of its applications has been significantly expanded [41, 42]. Some of the applications of this technique are bioreactor monitoring, environmental monitoring, air quality analysis, monitoring of metabolic processes, and determination of gases dissolved in water [41, 43]. Since the 2000s, the evolution of the MIMS technique was focused on the development of portable systems and on-site applications [42]. One portable MIMS system was used to develop a method for detecting the presence of people indoors, for security purposes, where the change of some analytes that are of interest for this research was monitored [44]. In addition, this technique was previously used for the exhaled breath analysis, but a different mass analyzer was employed, comparing to this study [40]. All the above supports this technique as a potential screening or diagnostic aid of the future, e.g., in human nutrition. In a recent review paper on volatilomics, performances of all the above-mentioned techniques are compared [45]. To get the best perspective, a comparison with GC–MS as a golden standard technique is the most relevant. Namely, it is known that GC–MS provides very high sensitivity at the ppt level. It is very successful in trace analysis, as it can offer preconcentration of analytes. Additionally, it provides an excellent selectivity and accuracy. It is used for qualitative and quantitative analysis, and it is equipped with rich commercial spectral libraries. However, it has some limitations that disable its wide and fast application. Bulky instrumentation, high costs, demand for laboratory conditions, and expert personnel are the points that could be improved. The MIMS technique is a good candidate to meet these limitations, for specific applications. It has been used for VOC analysis in gas, water, and soil matrixes. It provides good sensitivity at ppt-ppb levels, good selectivity, and qualitative and quantitative analyses. Unlike GC–MS which enables analysis only in the offline mode, MIMS can be used in the online mode as well. Moreover, it does not require laboratory conditions and it is easy to work with once the system is set. Duration of analysis is significantly shorter than with the conventional GC–MS. Main advantages are that no sample preparation is required and it has small dimensions, low power consumption, and low cost. However, MIMS has a limitation in complex mixture analysis, which is usually improved by coupling with a temperature-programmed desorption system [45]. MIMS surely cannot replace GC–MS, but it could be employed in specific areas of VOC analysis as a screening technique and to serve as a portable, affordable solution with good analytical performance.

## Materials and methods

### Sample collection

Exhaled breath samples were collected from 50 healthy adults—25 male and 25 female, who signed informed consent forms. Collection of samples was conducted at the BioSense Institute laboratory in Novi Sad, Serbia. Each participant provided 3 exhaled breath samples in single-use 1-L Tedlar® bags. The first sample was taken at 9 a.m., before breakfast, and after 12 h having restraint from food and beverages (only water was allowed). After providing the first sample, every participant ate a white flour pastry within 10 min. All participants ate the same pastry which was provided to them. The second and third breath samples were collected postprandial at 60 min and 120 min. Between the meal and the 3^rd^ sample collection, participants abstained from consuming anything (including cigarettes and chewing gums), except for pure water. Along with the samples, every participant filled a questionnaire which included questions about the gender and age, current living and working environment type, dietary habits, alcohol consumption and smoking habits, physical activities, and health condition. Questionnaires and samples were anonymized. Investigation was done in accordance with the current Serbian Data Protection Law (Official Gazette of the Republic of Serbia No. 87/2018), which follows the European *General Data Protection Regulation* since 2018, and with ethical approval from an independent ethical advisor, Prof. Gordana Vilotijević Dautović, who is an expert in medical ethics and a university professor in medical ethics and pediatrician pulmonology at the Medical Faculty of the University of Novi Sad (Approval No. 2021–01-3/70–1).

### Reagents and material

Reagents used in this research are commercially available chemicals in liquid phase, with purity grade > 99%. Used chemicals were methanol (Sigma-Aldrich, > 99.8%), ethanol (Honeywell, > 99.8%), acetone (Sigma-Aldrich, ≥ 99.5%), isoprene (Sigma-Aldrich, ≥ 99.5%), and n-pentane (Sigma-Aldrich, ≥ 99%). Parafilm M was also purchased from Sigma-Aldrich. Tedlar® bags with a capacity of 1 L were purchased from Zefon International. Nitrogen, argon, and krypton gases with purity 5.0 were purchased from Messer Tehnogas. A micropipette was purchased from IKA®-Werke GmbH & Co. KG. A SIL-TEC A class VI USP medical-grade polydimethylsiloxane (PDMS) sheet membrane was purchased from Technical Product Inc. of GA.

### Instrumentation

**Fig. 1:**
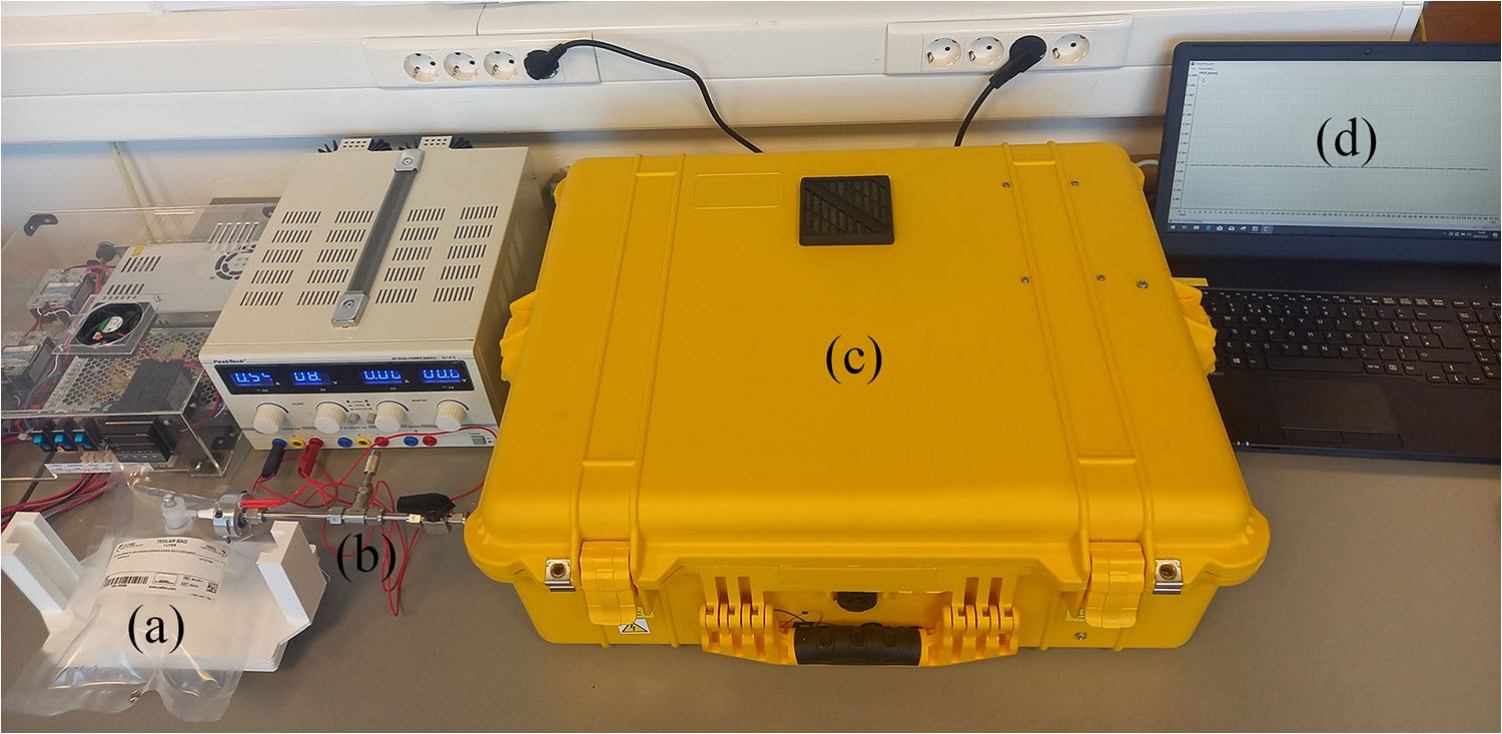
Exhaled breath sampling setup using a portable mass spectrometer coupled with a heated sheet membrane probe: (**a**) breath sample in the single-use sampling bag, (**b**) sheet membrane probe heated at 70 °C, (**c**) single quadrupole mass spectrometer, (**d**) laptop for acquisition and processing the results (Corel Graphics Suite 2021)

**Fig. 2:**
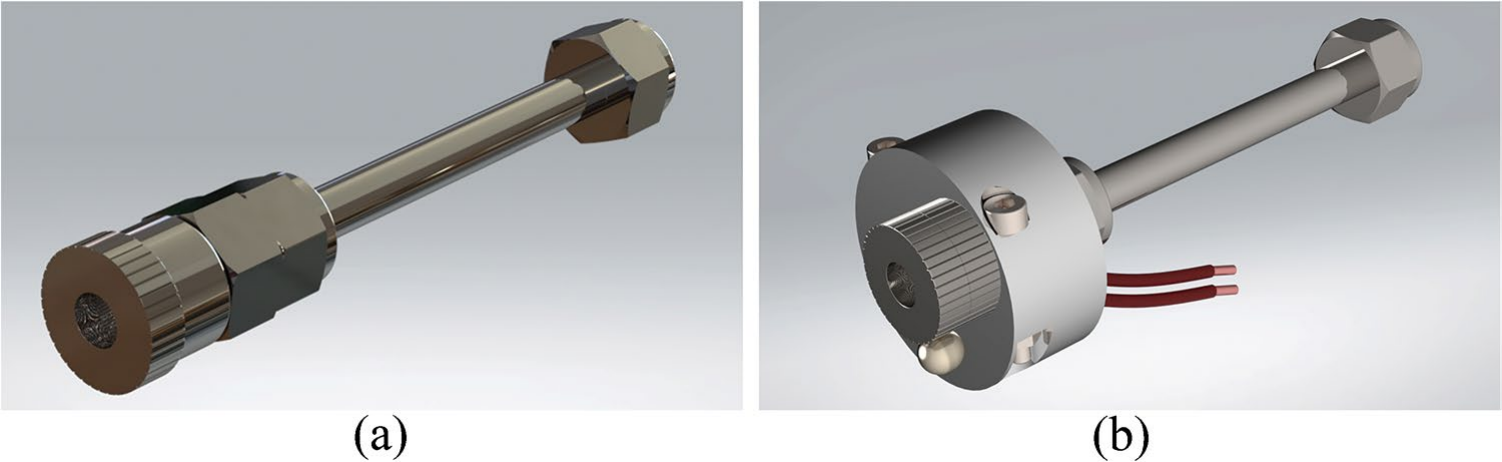
Schematic drawings of (**a**) the sheet membrane probe and (**b**) the sheet membrane probe with a stainless-steel assembly constructed with an integrated 12-V 50-W cartridge heater (SolidWorks, D.S., 2021, SP 5.1)

The portable mass spectrometry system employed in this study consisted of a membrane sample inlet, a single quadrupole mass spectrometer (QMS), a vacuum system (diaphragm and turbo pumps), and a laptop PC for data acquisition and processing of the results (Fig. 1). The QMS is a Prisma Plus® compact mass spectrometer (Pfeiffer Vacuum Gmbh) with a closed electron impact (EI) HS-gas tight ion source with yttriated iridium filaments, a 100-mm-long single quadrupole mass analyzer QMS 200 containing 6-mm-diameter round rods, a dual-detector (Faraday cup and secondary electron multiplier), and a suitable electronic control unit (ECU)—QME 220 M3. This mass spectrometer is designed for partial pressure analysis below 10^−4^ mbar. It provides unit resolution and fast response times (≤ 0.5 s) at low concentration levels (parts per billion) across the entire working mass range. In combination with its analytical mass range up to *m*/*z* 300, this system is optimal for determination of low-molecular-weight volatile compounds. The duration of one scan across the whole mass range is approximately 1 min, with a dwell time of 1 s. System dimensions are 616 × 220 × 433 mm (L × H × W) and it weighs 23 kg. Along with its low energy consumption (about 200 W/h at its maximum employment), real-time analysis capability, and scan speed, it is fully suitable for field work. This system was coupled with the sheet membrane probe (Fig. 2a). The stainless-steel probe was constructed “in-house” according to a previously reported design [44]. It was combined with a SIL-TEC A class VI USP medical-grade polydimethylsiloxane (PDMS) sheet membrane for selective introduction of VOCs to the system. This membrane can be used for a long time without replacement, as it is very durable. It is cleaned through heating and vacuum suction; thus, there is no danger of a carry-over effect [46]. The thickness of the membrane was 0.127 mm, and the active membrane area was 32 mm^2^. In order to increase sensitivity, the membrane was heated at 70 °C that was previously determined as the optimal temperature for this probe assembly [44]. Continuous heating was provided by a stainless-steel assembly constructed via CNC Mill (SYIL Machine Tools Co., Ltd.) with an integrated 12-V 50-W heating cartridge (Fig. 2b). A thermostat chamber (Memmert GmbH + Co.KG) was used for tempering the samples at 36–37 °C and for the calibration gas standard production at the same temperature, to correlate with normal human body temperature. Samples were introduced into the MIMS system by connecting the valve of the 1-L single-use Tedlar® bag with the sheet membrane inlet using a small PFA hose part, which was wrapped with a thermostable, inert Teflon® tape as shown in Fig. 1.

### Instrument tuning

The instrument was tuned prior to the analyses. The utilized quadrupole mass analyzer has an autotune option for Rf/DC voltage ramp and Rf frequency adjustment. Unit resolution, mass scale, and peak shape parameters were checked with nitrogen (*m*/*z* 28), argon (*m*/*z* 40), and krypton (*m*/*z* 84) pure gases, as their characteristic *m*/*z* values were suitable for examination of the analytical mass range relevant for this study. Furthermore, several ion source parameters were adjusted to enable an optimal sensitivity for the examined analytes (acetone, ethanol, isoprene, and n-pentane). This included optimization of the voltages applied to the filament and to the ion source electrode, which directly impacted the emission of the electron and their acceleration into the ion source, respectively. Voltage applied to the focus lens was adjusted to provide the optimal extraction of the ions from the ion source cage to the quadrupole mass analyzer, and voltage applied to the electron multiplier was adjusted for greater sensitivity. All of these parameters were optimized by analyzing the gas phase of the selected analytes.

### Calibration standard preparation

Gas standards for each analyte were prepared separately in adequate concentration ranges according to expected levels of selected VOCs in human breath based on the literature [3, 16, 47] and available technical possibilities: 10–1200 ppb for acetone, 10–500 ppb for ethanol, 5–100 ppb for n-pentane, and 25–200 ppb for isoprene. The preparation of calibration gas standards was conducted according to the static dilution method previously described by Naganowska-Nowak [48]. Liquid stock standard solutions for the selected VOCs were prepared in methanol. All solutions were freshly prepared at the day of analysis. Adequate volumes of working solutions were added with an automatic micropipette into a 1-L glass flask, which were immediately covered with several layers of parafilm. Closed flasks were conditioned at 36–37 °C in the thermostat chamber in order to mimic normal body temperature and to produce gas standards of selected volatile organic compounds in corresponding ppb concentrations. Laboratory atmospheric air was analyzed as a blank sample, in triplicate, in the beginning and in the end of the analyses.

### Method validation

The main subject of this research is the development of a bioanalytical method for determining the effect of food consumption on selected volatile compounds of human exhaled breath, using portable mass spectrometry with membrane injection.

In order to obtain validation of the bioanalytical method for monitoring exhaled breath VOCs, several parameters are evaluated: membrane inlet response time, linearity for the selected concentration range, sensitivity—limits of detection (LODs), and repeatability for the under-investigation analytes. All examined parameters are expressed via corresponding quantitative values. Inlet response time refers to rise and fall time for the measured ion current (IC) at a specific *m*/*z* value. This rise time was expressed as the time required to achieve an increase of the signal from 10 to 90% of the maximum ion current signal for each analyte, when the flask with the gas standard is set near the membrane inlet. The fall time was expressed as time required to achieve reduction in signal intensity from 90 to 10% of the maximum ion current signal after moving the flask with the gas standard from the membrane inlet. Examined LODs were in parts per billion per volume (ppb_v_) with 10 ppb for acetone and ethanol, 25 ppb for isoprene, and 5 ppb for n-pentane. For each analyte’s peak at the lowest concentration value (i.e., LOD), signal-to-noise ratio (S/N ratio) > 3 criteria was required. Linearity within the selected concentration range was expressed as a coefficient of determination (*R*^2^). Repeatability was defined as a relative standard deviation (RSD) among 3 measurements of the same breath sample, for selected *m*/*z* values. The average RSD values for examined analytes were calculated for all 150 analyzed exhaled breath samples. Repeatability at different days is calculated as well. The relative standard deviation (RSD) among measurements of the same calibration gas standard level on different days during a 5-month period was calculated. Average RSD values for all calibration levels were expressed for selected mass fragments.

### Food impact assessment

The determination of the timing for monitoring the largest change upon meal consumption was essential in order to establish the protocol for the VOC sensor method. A previously reported study in which acetone, ethanol, and isoprene levels upon meal consumption were examined for several time points (before the meal and 30 min, 60 min, 120 min, 180 min, 240 min, and 300 min after the meal), and our preliminary research with just 6 participants, narrowed our research to select to examine signals in the samples collected 60 min and 120 min after the meal.

Additionally, experimentally obtained relative responses for acetone, ethanol, n-pentane, and isoprene are compared against data collected from the questionnaire, in order to reveal possible correlations between levels of certain VOCs and their characteristics.

## Results and discussion

The NIST mass spectra library [49] was taken as a reference prior to the experimental phase for selection of the characteristic mass fragments. NIST data was confirmed experimentally by scanning pure gases for selected VOCs using our MIMS system. After both theoretical and experimental approaches, the most selective mass fragments for selected analytes were selected for qualitative and quantitative analyses. Characteristic mass fragments which were selected were *m*/*z* 42 for n-pentane, *m*/*z* 45 for ethanol, *m*/*z* 58 for acetone, and *m*/*z* 67 for isoprene.

### Method validation parameters

All calibration standards and all breath samples were monitored in the full-scan mode, in triplicates, and for selected characteristic *m*/*z* in the selected ion monitoring (SIM) mode. The SIM mode was used to determine response times (rise time and fall time) for selected analytes. During the rise time determination, once the maximum ion current signal is reached, that value is used for the construction of calibration curves, linearity determination, and the quantification of the VOCs in samples. Spectra in the full-scan mode were used for sensitivity (limits of detection) and repeatability determination. The summarized results are presented in Table 2, followed by explanations.

**Table 2:** Method validation parameter summary

VOC	Acetone	Ethanol	n-Pentane	Isoprene
Selected mass fragment (*m*/*z*)	58	45	42	67
Calibration curve range (ppb)	10–1200	10–500	5–100	25–200
Linearity (*R*^2^)	0.9969	0.9911	0.9815	0.9758
Calibration curve equation	*y* = 0.022*x* + 10.19	*y* = 1.52*x* + 424.12	*y* = 1.69*x* + 165.30	*y* = 0.048*x* + 5.43
Sensitivity, LOD (ppb)	10	10	5	25
Repeatability (RSD)	11^*^	11^*^	8^*^	13^*^
	29^**^	29^**^	24^**^	22^**^
Rise time, *t*_10–90%_ (s)	25^***^	50^***^	19^***^	27^***^
Fall time, *t*_90–10%_ (s)	33^***^	47^***^	51^***^	39^***^

^*^Repeatability calculated from the consecutive measurements of the same breath sample.

^**^Repeatability calculated from the relative intensities of the analyzed calibration gas standards at different days during a period of 5 months.

^***^Response times obtained in this study are longer than it is usual for membrane probes. This is due to the experimental setup, explained in the following text in detail.

#### Membrane response times

The response of the membrane was determined by calculating the rise and the fall times for the examined concentration levels of each analyte, which are presented in Table 2. The obtained signal rise times for acetone, ethanol, n-pentane, and isoprene were 25, 50, 19, and 27 s, respectively, while the obtained signal fall times were 33, 47, 51, and 39 s, respectively. Usually, the response time for the PDMS sheet membrane is quite short—few seconds [41]. However, in this experimental setup, the membrane probe was heated at 70 °C, so it was impossible to insert the probe directly through the parafilm into the glass flask, without melting the parafilm and losing the analytes. Thus, it was necessary to make some non-metal connection between the heated sheet membrane and VOC gas phase in the flask. A small PFA hose part wrapped with Teflon® tape was used for this purpose. Therefore, more time was needed for the analyte gas phase to reach the whole membrane area. From the results obtained, it can be concluded that ~ 1 min is sufficient for the membrane area to get saturated with the gas phase of the group of selected VOCs. In addition, about 1 min is needed after analysis to get the membrane ready for the next analysis. Considering that one scan across the mass range lasts about 1 min, it can be easily estimated that for one breath sample analysis using this VOC sensor, ~ 3 min is required. Nevertheless, the mass scan speed can be greatly improved by scanning the entire mass range within a second. This can be done by increasing the number of bits on an analog-to-digital converter (ADC) and using a high-speed amplifier in the quadrupole ECU.

#### Linear dynamic range within the examined concentration area

**Fig. 3:**
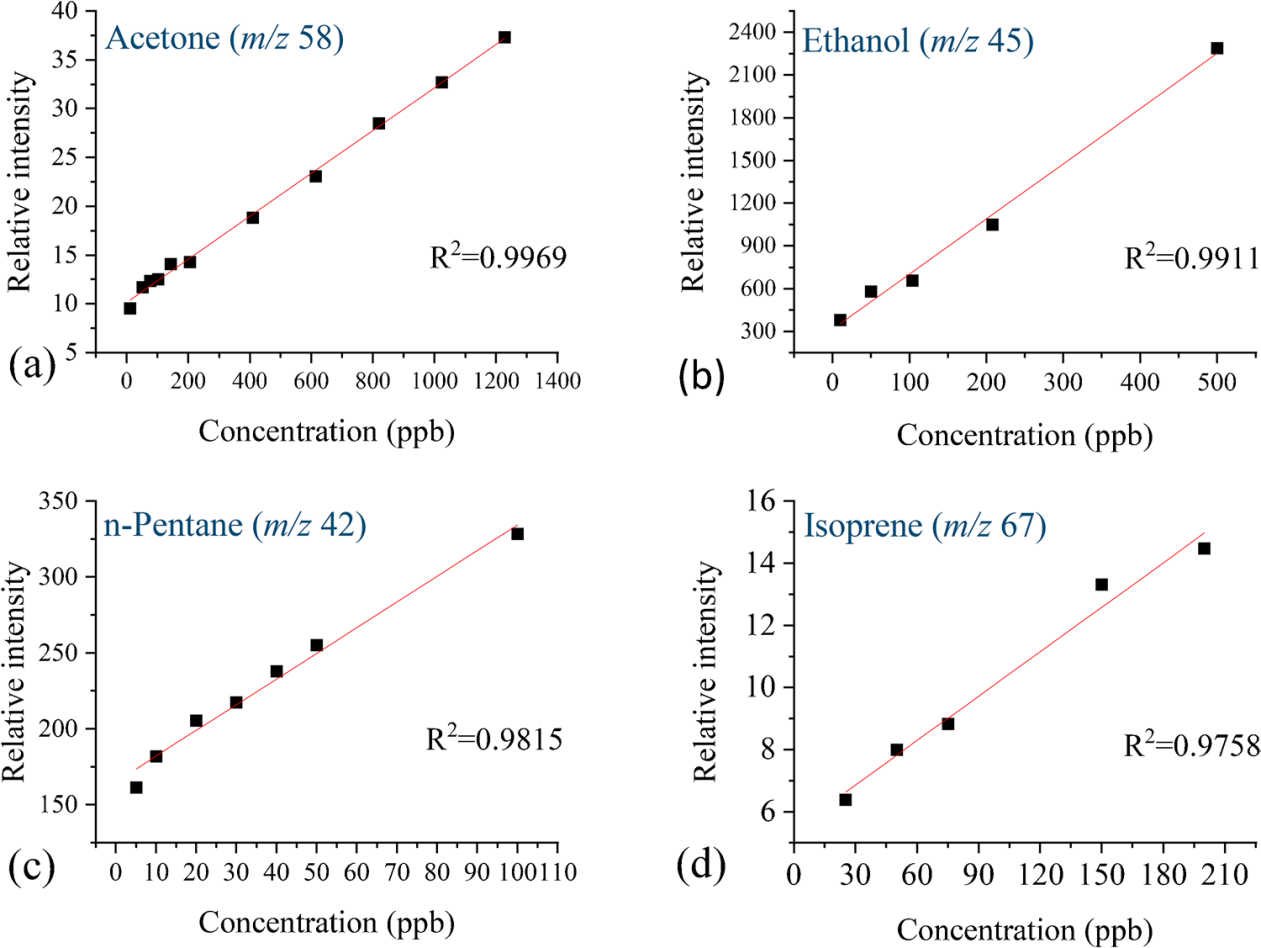
The calibration curves for the obtained concentrations for (**a**) acetone mass fragment *m*/*z* 58 ranging from 10 to 1200 ppb, (**b**) ethanol mass fragment *m*/*z* 45 ranging from 10 to 500 ppb, (**c**) n-pentane mass fragment *m*/*z* 42 ranging from 5 to 100 ppb, (**d**) isoprene mass fragment *m*/*z* 67 ranging from 25 to 200 ppb (OriginPro 2020b 9.7.5.184)

Calibration curves were constructed in adequate concentration ranges for each analyte: 10–1200 ppb for acetone, 10–500 ppb for ethanol, 5–100 ppb for n-pentane, and 25–200 ppb. At least 5 concentration levels are used for fitting the linear calibration curve using linear regression with the least square model (Fig. 3). Calibration curves’ equations and *R*^2^ values showing fairly good linearity for acetone (m/z 58), ethanol (m/z 45), n-pentane (m/z 42), and isoprene (m/z 67) are presented in Table 2.

#### Method sensitivity

The limit of detection (LOD) was taken as the minimum ion current signal for a certain m/z value which is 3 times higher than the ion current of the noise in the same spectra, i.e., the signal-to-noise ratio (S/N) must be higher than 3. Calculated S/N values for the lowest concentration levels for acetone (m/z 58), ethanol (m/z 45), n-pentane (m/z 42), and isoprene (m/z 67) are 15, 135, 44, and 4, respectively. Set criteria, S/N > 3, are satisfied for all analytes. Obtained LODs were 10 ppb for acetone (m/z 58) and ethanol (m/z 45), 25 ppb for isoprene (m/z 67), and 5 ppb for n-pentane (m/z 42) (Table 2).

#### Method repeatability

The relative standard deviation between 3 measurements for the same breath sample was calculated for each analyte’s ion current signal. Average RSD values for all 150 analyzed exhaled breath samples were 11% for acetone, 11% for ethanol, 8% for n-pentane, and 13% for isoprene (Table 2). Additionally, relative intensities obtained for all analytes’ calibration gas standards at different days during the 5-month period were used to express repeatability as well. The relative standard deviation for every calibration level was calculated, and average values for acetone, ethanol, n-pentane, and isoprene were 29%, 29%, 24%, and 22%, respectively (Table 2).

### VOC quantification

For the quantification of selected VOCs from breath samples collected before the meal, 60 min after the meal, and 120 min after the meal, an “in-house” Python application was developed. The application was programmed to generate ppb values for specific *m*/*z* values, based on the constructed and fitted linear calibration curves for each VOC. Considering that experiments were not conducted on the same day, relative intensities were generated, in order to enable comparison between all analyzed samples. Experimentally obtained maximal ion current (IC) values in amperes (A) for selected *m*/*z* values are normalized to maximal IC values for the *m*/*z* 14 (nitrogen) from the scan of the laboratory air at the same day when samples were analyzed. Relative intensities obtained by normalization were multiplied with a factor of 100,000 in order to facilitate the graphical representation. Comparing relative intensities for samples against constructed calibration curves, concentration levels in ppb for acetone (*m*/*z* 58), ethanol (*m*/*z* 45), n-pentane (*m*/*z* 42), and isoprene (*m*/*z* 67) for collected breath samples were obtained.

Mean concentration levels obtained for breath acetone (Table 3): 809 ppb BM, 875 ppb 60 min AM and 559 ppb 120 min AM with 95% confidence intervals 529–1189 BM, 629–1157 ppb 60 min AM and 393–773 ppb 120 min AM, were in the expected concentration range and in compliance with previously reported data. Acetone breath levels are normally in the range of 300–1000 ppb in the breath of healthy individuals, according to the recent comprehensive review of Das et al. [3]. Furthermore, Smith et al. [16] reported breath acetone levels determined from 30 individuals, for a 6-month period, to be in the concentration range 148–2744 ppb, and Diskin et al. [50] found that breath acetone is in range 293–870 ppb. The median concentration of acetone in healthy individuals is reported to be ~ 400 ppb in the non-starving condition, while during starving, it can be elevated up to 5–8 ppm [51]. Also, a study which involved 451 healthy individuals showed that the average acetone level was 450 ppb [52].

**Table 3:** The exhaled breath VOC results using MIMS

VOC	Before the meal (BM)		60 min after the meal (AM)		120 min after the meal (AM)	
	Mean (ppb)	95% confidence interval (ppb)	Mean (ppb)	95% confidence interval (ppb)	Mean (ppb)	95% confidence interval (ppb)
Acetone	809	529–1189	875	629–1157	559	393–773
Ethanol	488	396–602	532	415–684	474	383–589
n-Pentane	30	22–39	29	19–43	27	14–39
Isoprene	70	55–86	68	51–84	66	51–81

Mean concentration levels obtained for breath ethanol (Table 3) were 488 ppb BM, 532 ppb 60 min AM, and 474 ppb 120 min AM with 95% confidence intervals 396–602 BM, 415–684 ppb 60 min AM, and 383–589 ppb 120 min AM. Some of the previously reported levels of breath ethanol in healthy individuals were 27–153 ppb [50] and 0–1663 ppb with a mean value of 196 ppb [26]. Results in this research are somewhat higher which may be due to ethanol quantification for *m*/*z* 45, which is the mutual mass fragment of 1-propanol and 2-propanol. They can also be found in the breath in small concentrations and could have contributed to the estimated ethanol breath level. However, reported results are not unexpected since they are of the same order of magnitude as previously reported in the literature for healthy individuals.

Mean concentration levels obtained for breath n-pentane (Table 3) were 30 ppb BM, 29 ppb 60 min AM, and 27 ppb 120 min AM with 95% confidence intervals 22–39 BM, 19–43 ppb 60 min AM, and 14–39 ppb 120 min AM. It must be noted that for 76% of participants, the n-pentane level was below the method limit of detection (5 ppb). Thus, reported mean concentrations in Table 3 are extracted from a small number of participants (12) where concentration could be determined. If all values which were below LOD were considered as LOD/2 (i.e., 2.5 ppb), which can be done to avoid a decrease of data number, mean concentrations for n-pentane would be 9 ppb BM, 10 ppb 60 min AM, and 8 ppb 120 min AM. One recent study examined the difference in several breath VOCs among people who follow a vegan diet and those who follow a Mediterranean diet [53]. They discovered that n-pentane levels differ among these two groups of participants, and that people who follow the vegan diet have lower levels of n-pentane, possibly due to decreased oxidative stress as a consequence of a plant-based diet. Values reported for people who follow the Mediterranean diet (0.89–1.17 ppb) are more relevant for our study, as our participants reported to be omnivores. It can be observed that the levels in our study are greater, but still within the expected order of magnitude, according to the previous findings [3]. This difference could be caused by the different diet of participants in our study. Our participants reported to be omnivores, but there is no information about the amounts of meat consumption or quality of the diet, which was proved to have an impact on breath n-pentane level. Also, other factors such as lifestyle habits and environment which lead to oxidative stress may contribute to these differences. More investigation is needed to make a clear conclusion.

Mean concentration levels obtained for breath isoprene (Table 3): 70 ppb BM, 68 ppb 60 min AM, and 66 ppb 120 min AM with 95% confidence intervals 55–86 BM, 51–84 ppb 60 min AM, and 51–81 ppb 120 min AM were aligned with previously reported levels of isoprene in a healthy population. Namely, one study which involved 451 healthy individuals reported the average isoprene level to be 65 ppb [49]. Also, other studies reported the concentration level for isoprene to be ~ 100 ppb [3, 51]. Interestingly, one study examined the exhaled breath VOCs during exercising and reported that exhaled isoprene values were between 10 and 540 ppb, which is in accordance with our findings [54].

### Food impact assessment

**Fig. 4:**
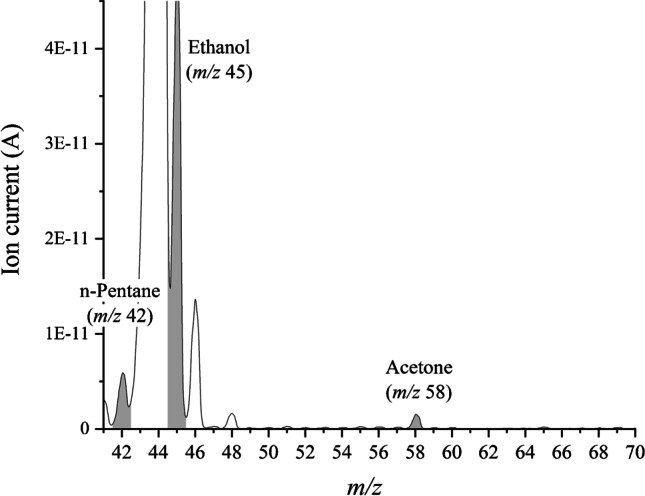
MIMS mass spectrum of the exhaled breath sample of a healthy non-smoker male participant who consumes alcohol moderately (2 drinks per week), practices physical activity 2–3 times per week (recreative-high category), and who lives and works in an urban environment (OriginPro 2020b 9.7.5.184)

The meal consumption impact on acetone (*m*/*z* 58), ethanol (*m*/*z* 45), n-pentane (*m*/*z* 42), and isoprene (*m*/*z* 67) levels in breath samples collected from 50 adult healthy participants (25 men and 25 women) was assessed by comparing the experimentally obtained results using the portable MIMS system at different time points—before the meal and 60 min and 120 min after the meal. A breath sample full scan from the one male participant is presented in Fig. 4.

The determination of food impact on selected breath VOCs and revealing the optimal time for monitoring the food impact on selected breath VOCs considered comparison of relative responses obtained in samples before and after the meal. Relative responses obtained in samples collected 60 min and 120 min after the meal were divided by relative responses in samples collected before the meal. Calculated ratios, called comparison factors, were used to determine if there is a change upon meal consumption at selected time points or not. A comparison factor with a value of 1 represents that there was no change upon food consumption. A significant change was considered when 10% of the VOC signal change upon meal consumption occurred, and it was observed in ~ 85% of participants for acetone and ethanol, ~ 70% for n-pentane, and ~ 50% for isoprene. Moreover, the intensity of the impact was evaluated at two time points, and it was determined that for acetone, n-pentane, and isoprene, there were more significant signal changes among samples collected 120 min after the meal, while for ethanol, more significant changes were observed in samples collected 60 min after the meal.

To our best knowledge, only few studies tried to monitor food impact on breath VOCs, so far. The impacts of different types of meals were examined. One example is a pilot study in which the metabolic effect of dietary fiber on exhaled breath was examined [18]. This study employed the SPME–GC–MS technique to monitor levels of 15 breath VOCs in 7 healthy men, upon consuming a high-fiber meal and a low-fiber meal. Among 15 examined VOCs, acetone and ethanol results are of interest to be compared with the results of this study. The postprandial acetone level decreased after both high-fiber and low-fiber meals. However, the high-fiber meal caused more significant change at all time points, while the low-fiber meal, which is more like the meal tested in our research, expressed its effect only 120 min after the meal consumption, which is supported by our findings. Furthermore, we agree on the ethanol level behavior upon meal consumption as well. They reported that the ethanol level increased after 30 min for both high-fiber and low-fiber meals, which is partly supported by our findings as we observed a greater change in ethanol level 60 min AM, rather than at 120 min AM, and we noticed an increase in ethanol signal as well. Moreover, levels of 5 different breath VOCs among which were acetone, ethanol, and isoprene were examined before the meal and after the consumption of a protein-calorie meal at several time points in the study of Smith et al. [16]. According to this study, acetone concentration was highest before the meal (after 12 h of fasting) with concentrations of ~ 200–600 ppb and it reached the greatest decrement after 4.5 h and a concentration ~ 200 ppb, although a significant signal decrease could be noticed even after 2 h [16]. Ethanol showed an increment upon meal consumption—from 50 to 100 ppb measured before the meal up to 100–400 ppb at the highest concentration 1 h after the meal [16]. In a previously reported study, isoprene showed a slight increment within 30 min after the meal [16]. Our results are aligned with this study due to observed changes in time for acetone and ethanol levels upon food consumption. Isoprene changes were not significant in both studies. Quantified levels are slightly higher in our study, but considering the diversity of biological systems and the number of factors which can interfere, we believe that it could be said that our results are supporting previously reported work.

### Correlations between VOC levels and participants’ lifestyle

**Fig. 5:**
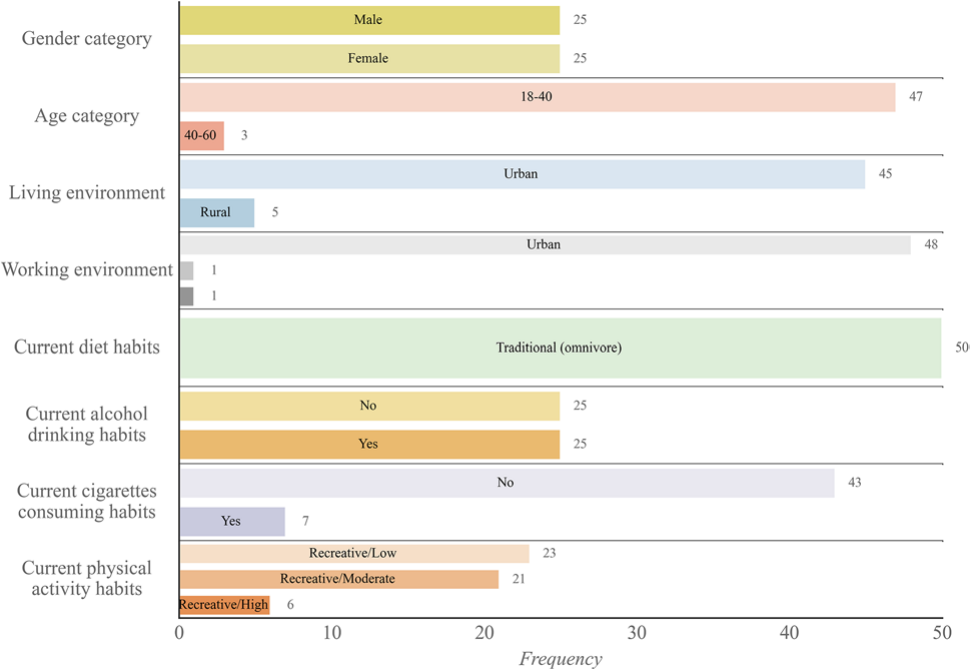
Bar plot with evaluated participants’ data collected via questionnaire (Python 3.9.12)

In general, two types of variables were collected within this study—numerical and categorical. Numerical variables were processed sets of measurements on samples for selected breath VOCs (comparison factors and ppb levels), while categorical variables were information about participants collected through the questionnaire: age and gender categories, living and working environment types, cigarettes and alcohol consumption, and dietary habits. The number of participants were deployed for each categorical parameter, and an evaluation of the examined population is presented in Fig. 5.

After questionnaire data evaluation, it can be said that some of the categorical parameters were quite homogeneous—participants were predominantly within the same age category (18–40 years old), living and working in the urban environment, and following an omnivore diet. Thus, these categorical parameters were not considered as variables.

**Fig. 6:**
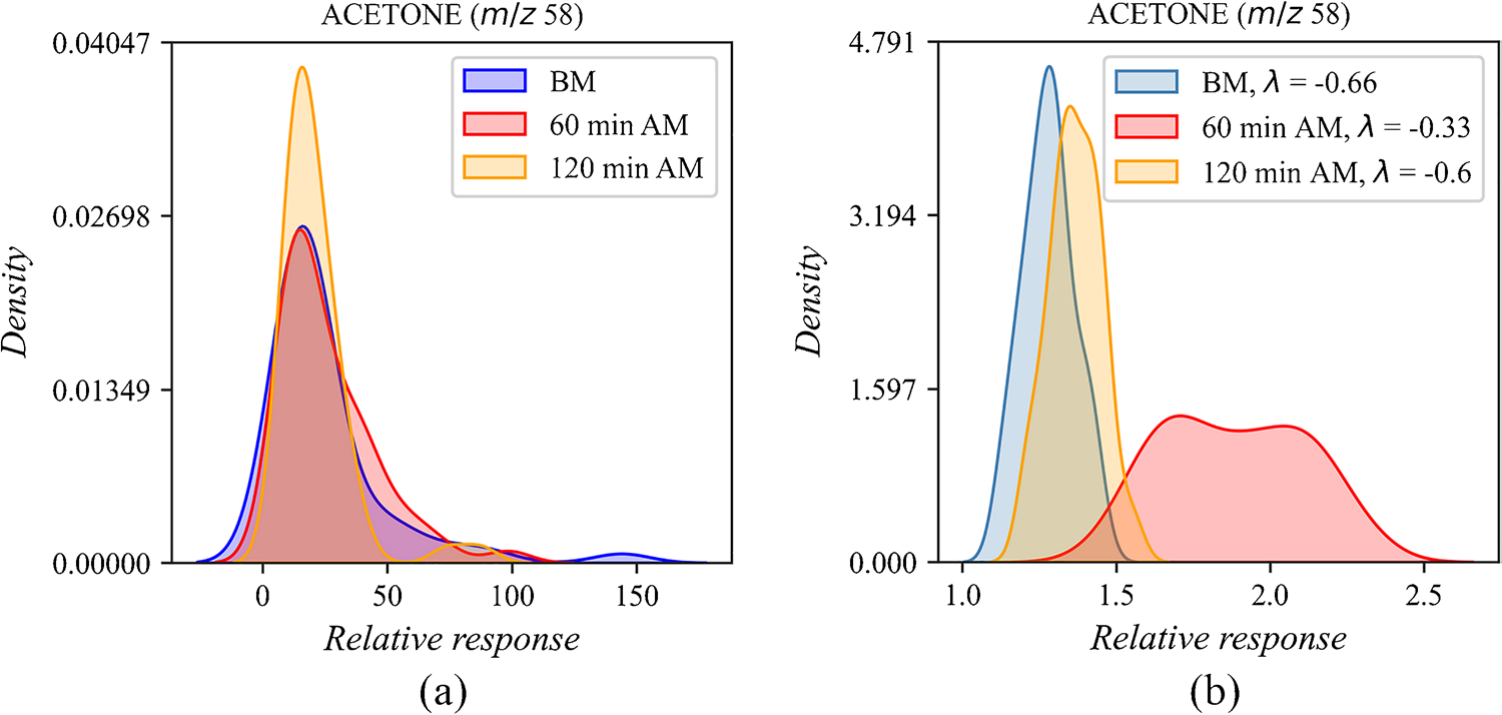
(**a**) Kernel density estimate (KED) plot after Shapiro–Wilk test for acetone detected in 150 exhaled breath samples; (**b**) kernel density estimate (KED) plot for acetone in 150 exhaled breath samples after Box-Cox transformation to normal distribution (Python 3.9.12 and Corel Graphics Suite 2021)

For discovering possible correlations between breath VOC levels and participants’ characteristics and lifestyle habits, statistical tests are used. The Shapiro–Wilk test [55] indicated that the distribution of the samples was not normal or log-normal (Fig. 6a). However, a tendency for normal distribution was noticed. Assuming that with a larger cohort of participants a normal sample distribution would emerge, a Box-Cox transformation [56] was applied to the current samples (Fig. 6b). This transformation to a normal distribution enabled the application of a one-way ANOVA test for finding possible correlations.

The one-way ANOVA test in this study was used twofold. Firstly, it was used for discovering possible correlations between VOC level behavior upon meal consumption, using a comparison factor from samples collected 120 min AM and specific gender or lifestyle habits (Table 4). Comparison factors represent the ratio of a specific VOC signal obtained from samples after the meal and that from samples before the meal. As mentioned previously, this factor tells if there was a change upon the meal in the VOC signal or not. If there is no change upon the meal, this ratio would be 1, while if the change did occur, the ratio would be different from 1. According to how it is different from 1, it tells if the VOC signal increased after the meal (if the ratio is higher than 1) or decreased (if the ratio is less than 1). Whether there were differences in some categories of participants that showed similar trends in VOC signal response to the food, a one-way ANOVA *p* value would indicate correlation. Secondly, it was used to see if there was any significant relationship between VOC mean values and categorical parameters (Table 5). For this purpose, 120-min postprandial mean values were used. It must be emphasized that n-pentane concentrations were below LOD (5 ppb) in more than half of the samples. Thus, in order not to reduce the amount of data in the statistical test, values which were below LOD were considered as LOD/2 (i.e., 2.5 ppb). A significant difference between values for two examined parameters was considered when the obtained *p* value was less than 0.05. Tables 4 and 5 summarize the obtained *p* values for all analytes.

**Table 4:** One-way ANOVA *p* values obtained using comparison factors for acetone, ethanol, n-pentane, and isoprene against the information collected via questionnaire from the same participants

Categorical parameter		Acetone	Ethanol	n-Pentane	Isoprene
Gender	Male vs. female	0.938	0.214	0.076	0.356
Alcohol drinking habits	Yes vs. no	**0.029**	0.725	0.598	0.682
Cigarette consumption habit	Yes vs. no	0.873	0.423	0.129	0.117
Physical activity habits	Low vs. moderate vs. high	0.747	0.331	0.136	0.718

Bolded *p*-values are those below the one-way ANOVA test treshold (α=0.05), implying statistically significant difference between breath VOCs changes observed among examined groups of the respective categorical parameter

**Table 5:** One-way ANOVA p values obtained by comparing VOC levels in samples 120 min AM and categorical parameters

Categorical parameter		Acetone	Ethanol	n-Pentane	Isoprene
Gender	Male vs. female	0.438	0.348	0.083	0.743
Alcohol drinking habits	Yes vs. no	0.24	0.673	0.79	0.738
Cigarette consumption habit	Yes vs. no	**0.017**	0.628	0.712	0.707
Physical activity habits	Low vs. moderate vs. high	0.605	0.327	0.093	0.089

Bolded *p*-values are those below the one-way ANOVA test treshold (α=0.05), implying statistically significant difference between breath VOCs changes observed among examined groups of the respective categorical parameter

One-way ANOVA *p* values obtained by comparing 120 min AM comparison factors for examined VOCs showed that among this group of participants, there are significant differences only in acetone levels between participants who consume alcoholic beverages and those who do not (i.e., *p* = 0.029, Table 4). Of course, this is a very small number of participants, and we believe that with an increasing number of participants in a future research, it will be possible to find more correlations. Previously, food intake impact on acetone level was examined [57]. That study reported that the acetone level is affected more by physiological factors rather than diet. However, the study involved only 30 participants, which could be a small cohort to reveal the dietary impact although they did report that participants who consumed low-calorie meals had higher acetone levels than those who ate more caloric food. Surely, more research in this area is needed.

By applying the one-way ANOVA test on ppb values for examined VOCs and the same categorical parameters, it can be concluded that there is significant difference in the levels of acetone between participants who consume cigarettes and those who do not (i.e., *p* = 0.017, Table 5). The relationship between smoking and increased breath acetone was reported in one recent study by Zhang et al. [58]. As the statistical test did not show many correlations, we assumed that it may not be sensitive enough for this number of participants. Thus, in addition to one-way ANOVA tests, we performed a simple categorization of the obtained mean values according to the data collected via questionnaire (Table 6). Higher levels between examined categories are highlighted due to easier observation of possible correlations.

**Table 6:** Mean values for acetone, ethanol, n-pentane, and isoprene by participant categories

Categorical parameter	Category	Acetone (ppb)		Ethanol (ppb)		n-Pentane (ppb)		Isoprene (ppb)	
		BM	AM	BM	AM	BM	AM	BM	AM
Gender	Female	716	509	441	386	*44*	13	63	62
	Male	*917*	*622*	*534*	*563*	25	*32*	*78*	*69*
Cigarette consumption habit	No	646	500	471	*491*	25	*30*	66	65
	Yes	*2400*	*1037*	*594*	373	*53*	13	*93*	*71*
Alcohol consumption habit	No	778	350	443	*541*	23	*39*	62	60
	Yes	*845*	*779*	*536*	407	*38*	16	*76*	*70*
Physical activity habits	Low	*891*	*593*	*495*	*589*	*31*	*36*	64	64
	Moderate	763	433	421	368	30	11	*76*	*67*

The higher breath VOCs levels obtained among examined groups for each categorical parameter are highlighted

It can be observed that single correlation revealed by ANOVA test can be confirmed by this categorization of data, as participants who consume cigarettes obviously have a higher level of acetone. Also, we noted that the isoprene level is slightly increased in the same category of participants. Regarding participants who consume alcohol, higher levels of acetone and isoprene are noticed as well. For physical activity impact, it was interesting to monitor the isoprene level, as it was previously discovered that it is increased upon physical activity [59]. However, it was reported that this impact can be observed only right after the activity. From Table 6, it can be seen that there is a slightly higher level of isoprene in participants who exercise more often, but it is not statistically significant. Future studies will reveal the possible long-term effect of exercise on breath isoprene. Gender impact was observed only for acetone level, although slightly higher levels of ethanol and isoprene were reported in male participants. Correlations between gender and acetone breath level were examined in the study of Turner et al. [57]. They found that there were significant differences in acetone levels between genders, which confirms our assumption. The same group of scientists examined the impact of gender on isoprene levels, and they did not find significant correlations, which is supported by our findings [59]. No significant correlations were observed for ethanol and n-pentane levels and examined categorical parameters. A recent study revealed that there is a statistically significant difference in breath n-pentane level between people with two different diet types—Mediterranean and vegan [53]. Unfortunately, in our study, it was not possible to inspect that kind of impact, as all participants reported to be omnivores. Further research will include more people with different diet types, and hopefully reveal more information regarding food impact on selected breath VOCs.

## Conclusions

The development of a new bioanalytical method in the exhaled breath VOC field is a very challenging quest. The exhaled breath is a very unstable analytical matrix, as there is a number of variables which contribute to it in every moment, i.e., biological and environmental factors. It can be said that even conventional techniques are not capable of giving consistent results. However, this should not be discouraging since great diagnostic potentials lie in this research area. As there are more studies involved, there are more confirmations of previous findings and significant knowledge emerges. Since technological possibilities are nowadays at a high level, we can simultaneously work on discovering new breath biomarkers and on making those screening tests more affordable and widespread. Thus, new analytical approaches which could give fast and reliable results, have smaller costs, and give real-time and portable solutions are highly valued.

The MIMS system used and presented in this study is more affordable than a conventional gas chromatograph coupled to a mass spectrometer, which is otherwise used for the analysis of VOCs in human exhaled breath. Compared to the conventional technique, the performance of the proposed screening sensor is partially limited, but on the other hand, it does not require strict laboratory conditions due to its portability. Therefore, its application could be far more widespread, which is a great advantage in achieving preventive screening analyses that would provide framework guidelines and signal the need for further, more detailed analyses.

In order to establish the new bioanalytical method for breath VOC detection using the portable MIMS system for the first time in food impact assessment, several parameters for confirmation of its functionality were examined. Adequate sensitivity and very good linearity across the examined concentration ranges were obtained for acetone, ethanol, n-pentane, and isoprene. Additionally, membrane response time was up to 1 min for all analytes, and the duration of one scan across the working range (*m*/*z* 35–200) was ~ 1 min, indicating that the estimated time for the analysis of one breath sample is about 3 min, which is much faster than the analytical run by the conventional technique.

This research work showed that this method can be applied for the determination of breath acetone, ethanol, n-pentane, and isoprene concentration at the ppb level. It cannot be said with what accuracy, as spiking of samples or internal standard utilization were not applicable. However, considering previously reported results, it can be mentioned that concentration levels estimated in this research are well aligned and that this method can be employed for breath VOC analysis as the screening sensor technique and to provide indicative levels for examined breath VOCs.

After setting the method, food impact assessment experiments were conducted. Considering all the results obtained—the percentage of participants where significant food impact was noticed, the intensity of that impact at 3 time points, and the concentration levels obtained for selected VOCs—this study shows that the greatest food impact was observed for the acetone breath level 120 min after the meal. This conclusion is expected and supports previous findings about food impact on breath acetone. In addition, the provided meal was high in carbohydrate content and, among all examined VOCs, the most relevant to reflect food impact was acetone due to its well-known relation to glucose level and carbohydrate metabolism. Furthermore, as it was previously reported and confirmed experimentally in this study, the ethanol level was increased in the first 60 min upon the meal and that time point would be optimal to examine the food impact at this VOC. It was not previously known what impact such a meal would reflect on n-pentane and isoprene levels. As it is known that these two VOCs are related to fat metabolism and n-pentane is related to antioxidant status, it was expected that their levels will not get highly affected by the carbohydrate meal, although the slightly greater change was noticed 120 min after the meal. Keeping in mind that fat metabolism is generally slower than carbohydrate metabolism in humans and that a significant food impact at the acetone level was observed 120 min after the meal, it was concluded that the food impact on this group of breath VOCs should be monitored 120 min after the meal.

Future research will include meals with mixed carbohydrate, fat, and protein content, and we expect to reveal greater reflection of food on all examined breath VOCs. One-way ANOVA statistical test was conducted in order to reveal possible correlations between obtained VOC levels and participants’ lifestyle habits. At this stage of the research, we could not reveal many correlations, but future research will involve more healthy participants with different diet and lifestyle habits, but also ones dealing with overweightness, obesity, diabetes, and cardiovascular difficulties. We believe that this investigation will lead to more relevant evaluation of the food impact on breath VOCs, but it will also provide insights into possible relations between physical activity, diet type, etc., and specific breath VOCs.
